# Long Haulers—What Is the Evidence for Post-COVID Fatigue?

**DOI:** 10.3389/fpsyt.2021.677934

**Published:** 2021-05-21

**Authors:** Andreas Stengel, Nisar Malek, Stephan Zipfel, Siri Goepel

**Affiliations:** ^1^Department of Psychosomatic Medicine and Psychotherapy, University Hospital Tübingen, Tübingen, Germany; ^2^Department for Psychosomatic Medicine, Charité Center for Internal Medicine and Dermatology, Corporate Member of Freie Universität Berlin, Berlin Institute of Health, Charité - Universitätsmedizin Berlin, Humboldt-Universität zu Berlin, Berlin, Germany; ^3^Department of Internal Medicine 1, University Hospital Tübingen, Tübingen, Germany; ^4^German Center for Infection Research (DZIF), Clinical Research Unit for Healthcare Associated Infections, Tübingen, Germany

**Keywords:** anxiety, chronic COVID, depression, functional, long covid, post-infectious, psychosomatic, post-acute sequelae of SARS-CoV-2 infection (PASC)

## Introduction

Since the start of the pandemic until now (end of February 2021), more than 113 million people worldwide have been infected (encompassing people with and without symptoms) with SARS-CoV-2, with 89 million of those (78.8%) being classified as recovered (1). However, it became more and more apparent that people infected can have symptoms not only in the acute phase but also with a considerable delay. A recent meta-analysis from 15 studies encompassing 47,910 patients showed that 80% of patients develop at least one symptom during follow–up times ranging from 2 weeks to 4 months post viral infection (2). This condition is termed heterogeneously, namely, long COVID, post-COVID, or recently post-acute sequelae of SARS-CoV2 (PASC). The most common symptoms were fatigue (58%), headache (44%), attention disorder (27%), hair loss (25%), and dyspnea (24%) (Table 1) (2). The long-term outcome of these symptoms remains to be investigated. The current opinion article will discuss the current state of knowledge on the development of long COVID symptoms with a special focus on post-COVID fatigue being the most common symptom.

**Table 1:** Common symptoms of long COVID.

**Symptom**	**Percentage**
Fatigue	58
Headache	44
Attention disorder	27
Hair loss	25
Dyspnea	24
Ageusia	23
Anosmia	21
Polypnea	21
Cough	19
Joint pain	19
Sweating	17
Memory loss	16
Nausea	16
Chest pain/discomfort	16
Hearing loss/tinnitus	15
Anxiety	13
Depression	12
Digestive disorders	12
Cutaneous signs	12
Weight loss	12
Sleep disorder	11
Fever	11
Pain	11
Resting heart rate increase	11
Palpitations	11

*Data are derived from Lopez-Leon et al. (2). Data are derived from 15 studies encompassing 47,910 patients*.

## Discussion

Early on, it was reported that patients after hospitalization due to SARS-CoV-2 infection may develop persistent breathlessness (53%) and cough (34%) during the following months post discharge (3). Among those patients, one third had persistently elevated d-dimer levels; in 10% of patients, the C-reactive protein levels were elevated (3), giving rise to an ongoing inflammatory reaction. This was also reflected in a high percentage (38%) of pathological chest x-rays likely due to active endothelitis/vasculitis as recently discussed (4). Several risk factors for the development of these persistent symptoms have been identified: besides the severity of the initial SARS-CoV-2 infection, higher age, higher body mass index, and pre-existing lung diseases were associated with a greater risk (5).

Interestingly, the majority of these patients described above also reported other symptoms. Two thirds (69%) also complained about fatigue; another 13 and 12% patients showed signs of anxiety and depression, respectively (3). However, another publication showed that fatigue developed irrespective of a preceding hospitalization and was therefore not associated with the severity of the initial SARS-CoV-2 disease (6). Moreover, female sex (7) and previous episodes of depression or anxiety were observed more often in patients developing fatigue (6), which contrasts with the risk factors for the development of the vascular long COVID symptoms. A perceived high level of perceived social stigmatization due to the infection status was associated with an increased risk of developing impaired general mental health post SARS-CoV-2 infection, while a high level of social support showed the opposite association (8).

Patients with fatigue did not show differences in autonomic functions (assessed using electrocardiogram and blood pressure monitoring during deep breathing, active standing, Valsalva maneuver, and cold-pressor testing) compared to subjects that underwent a SARS-CoV-2 infection without developing fatigue (9). This gives rise to a functional origin of the fatigue symptoms.

It is well-known that irritable bowel syndrome (IBS), a highly prevalent (worldwide prevalence around 10–15%) functional gastrointestinal disorder (10), can develop after acute gastroenteritis, also known as post-infectious IBS (11). Symptoms often encompass diarrhea and abdominal pain, being not different from those of a non-post-infectious genesis of IBS (10). Likewise, the treatment does not differ as no persistent inflammatory changes are present that would explain the magnitude of complaints (10). Therefore, also these complaints are classified as functional. The current pathogenetic model explaining the pathogenesis of functional disorders is the biopsychosocial model encompassing biological (e.g., genetic factors) and psycho-social (e.g., life events, acute triggering somatic, or psychological stressors) factors (12). According to the new International Classification of Diseases 11th revision (ICD-11), this will be best categorized as bodily distress disorder, “characterized by the presence of bodily symptoms that are distressing to the individual and excessive attention directed toward the symptoms which may be manifest by repeated contact with health care providers” (13). “Typically, the disorder involves multiple bodily symptoms that may vary over time. Occasionally there is a single symptom—usually pain or fatigue—that is associated with the other features of the disorder” (13).

In summary, a SARS-CoV-2-infection might trigger post-COVID fatigue as a variant of long COVID. Whether this is specific for SARS-CoV-2 or rather a broader risk factor for the development of a functional disease/bodily distress disorder as observed before for, e.g., post-infectious IBS, will have to be further investigated. Moreover, more emphasis should be given to the characterization of risk factors for the development of functional post-COVID symptoms so patients at higher risk can be given higher attention in a personalized approach, screened early, and provided early help to avoid over-diagnosing and iatrogenic somatic fixation. Hereby, the close collaboration of internal medicine practitioners and those specialized in psychosomatic medicine/psychiatry is key to offer treatment in an integrated manner. It is likely that treatments encouraging active participation of patients (e.g., exercise or psychotherapy) might be more effective than passive options (e.g., medication alone) as described for other functional disorders before (14).

## Author Contributions

AS prepared the first draft of the article. All authors critically reviewed and finalized the opinion article.

## Conflict of Interest

The authors declare that the research was conducted in the absence of any commercial or financial relationships that could be construed as a potential conflict of interest.
